# Effects of high-molecular-weight glutenin subunit on hard-steamed bread quality

**DOI:** 10.3389/fgene.2024.1473518

**Published:** 2024-10-03

**Authors:** Jing Zhao, Tianyi Wang, Jiajia Zhao, Ling Qiao, Bangbang Wu, Yuqiong Hao, Chuan Ge, Zhiwei Feng, Xingwei Zheng

**Affiliations:** ^1^ Institute of Wheat Research, Key Laboratory of Sustainable Dryland Agriculture (Co-construction by Ministry and Province) Ministry of Agriculture and Rural Affairs Shanxi Agricultural University, Linfen, China; ^2^ College of Agriculture, Shanxi Agricultural University, Jinzhong, China

**Keywords:** HMW-GS, steamed bread, subunit, physical property indexes, dough rheological properties

## Abstract

**Introduction:**

Steamed bread (SB) is a daily food in many countries in the world, but the relationship between HMW-GS and the quality of SB remain unclear.

**Methods:**

This study investigated the effects of 12 subunit combinations on the characteristics of SB, including volume, physical properties, and sensory evaluation, combined with the microstructure and dough rheological properties.

**Results:**

The locus effect results showed, volume and physical properties of SB were *Glu-D1*>*Glu-B1*>*Glu-A1*, while sensory scores *Glu-B1*>*Glu-D1*>*Glu-A1*. According to individual subunit effects, subunit 1 at *Glu-A1* locus, 7+8 and 7+9 subunits at *Glu-B1* locus, and 2+10 and 5+12 subunits at *Glu-D1* locus were significantly superior to other subunits in physical indices like volume, chewiness, glueyness, and sensory scores, and were less affected by moisture. The effect of subunits combination is mainly affected by subunits, and the combination of superior subunits tends to make SB quality better. The subunit combinations (1, 7+8, 5+12), (N, 7+9, 2+10) and (1, 7+9, 5+12) had better physical properties indexes, sensory scores, dense, uniform and delicate micro-pore structure, and smaller thickness wall.

**Discussion:**

The results showed that protein content, wet gluten content and stability time were the main factors affecting the volume and physical properties of SB. The protein content, wet gluten content and stability time of flour in 7+8, 7+9, 2+10 and 5+12 subunits were higher. Therefore, the quality of SB containing these subunits was found better.

## 1 Introduction

Steamed bread is a daily food in many countries worldwide, especially in Europe and Asia. In Germany, it has been called “Dampfnudel,” in Russia, it is “pot lid bread,” and in the United Kingdom, “steamed dumplings.” Japan, North Korea, and South Korea use the term “steamed bread,” and it is a traditional staple food in China. More than 1.8 million tons of common wheat are used to produce steamed bread yearly (Fan et al., 2009). According to taste preference, it can be classified as either hard or soft steamed bread and subdivided further into either traditional sourdough- or yeast-steamed bread (Huang et al., 1993). Hard-steamed bread is chewy, with relatively greater weight, volume, and density, and with a smaller and more elastic internal mesh. In contrast, soft-steamed bread is softer, smaller, and lighter and has a larger internal mesh (Huang et al., 1996). The flour for making steamed bread comes from wheat with medium to medium-strong gluten, protein content ≥12.5%, 28%–33% wet gluten content, and a formation and stabilization time of 3–6 min (Chinese National Standard GB/T 17320-2013). Whether it is hard- or soft-steamed bread, the requirements for dough rheology and fermentation process are almost the same, although the requirements for kneading moisture are different. As standards of living have increased, so has the demand for higher-quality steamed bread products. The content and strength of gluten are the main factors that influence the volume, surface structure, and internal structure of steamed bread, and glutenin is an essential component of wheat gluten (Xie et al., 2023). Therefore, studying glutenin and wheat processing characteristics can provide a basis for analyzing steamed bread quality and breeding varieties.

Glutenins comprise high- and low-molecular-weight glutenin subunits (HMW-GSs and LMW-GSs), which form gluten macropolymers stabilized by interchain disulfide and hydrogen bonds. Although HMW-GSs only account for 10% of total proteins, they explain approximately 45–70% of the variation in processing quality (Payne et al., 1981; Lawrence and Shepherd, 1981; He et al., 2005). HWM-GSs are encoded by genes located on the long arms of chromosomes 1A, 1B, and 1D. Each locus has two closely linked genes controlling the x- and y-type subunits, and the variation generated by different combinations of these loci affects wheat flour products and processing quality (Zhang et al., 2007). At present, the influence of HMW-GSs on the processing quality of bread, noodles, and biscuits is relatively well-understood (Carrillo et al., 1990; Li et al., 2015; Yang et al., 2023), and the baking quality of biscuits and bread with subunits 17+18 and 5+10 is higher (Brönneke et al., 2000; Sharma et al., 2022). This subunit combination appears more frequently in European hexaploid wheat. Zhu et al. (2001) showed that the total rating of steamed bread was greater for the subunit 5+10 versus 2+12, but Fan et al. (2005) recorded that the subunit 5+10 had the undesirable effect of increasing product stickiness. Little research has been done to quantitatively analyze the effects of different subunits on steamed bread quality. There are 16 types of common HMW-GSs, such as 1, N, 6+8, 7+8, 5+10, 5+12, etc. At present, only eight kinds of subunits, such as 1, N, 7+8, 7+9, 2+12, and 5+10, have been reported (Fan et al., 2005). Previous studies on the relationship between HMW-GSs and the quality of steamed bread used only a few varieties, and the difference in genetic background led to inconsistent evaluation of the effects of HMW-GSs. Such inconsistencies could be reduced by using near-isogenic lines (NILs), recombinant inbred lines (RILs), and double haploid (DH) materials with specific HMW-GS differences by assessing genetically diverse lines with the same HMW-GSs and by eliminating background influences from LMW-GSs, gliadin, starch, and lipids (Nieto-Taladriz et al., 1994; Popineau et al., 1994). Therefore, studying the effects of a larger number of subunits on steamed bread quality through these types of studies could provide a stronger theoretical basis for the selection and quality prediction of special steamed bread varieties.

Moisture is the most significant factor affecting the growth of wheat. Protein content is often negatively correlated with rainfall and soil moisture content, and excessive moisture impacts protein content and gluten elasticity, thus affecting the baking quality of bread (Campbell et al., 1981; Zhao et al., 2020). However, the effects of HMW-GSs on flour and steamed bread quality from wheat grown under different moisture conditions have not been studied. According to Wang et al. (2004), when wheat was irrigated once or twice post-anthesis, the resulting harvest produced flour with increased dough formation and stabilization time, but these qualities decreased with further irrigation. Thus, irrigation schemes should be formulated according to the quality requirements of the final product being targeted (Guttieri et al., 2000; Guttieri et al., 2001). In the present study, four representative parents were used to construct the DH population to study the effects of HMW-GSs on the quality of steamed bread produced from wheat crops grown under different moisture conditions, and the relevant reasons were analyzed from the rheological characteristics of dough. The results of the present study can provide references for the quality breeding and cultivation measures of different ecological types of wheat.

## 2 Materials and methods

### 2.1 Plant materials

In total, 469 lines of a DH population were derived from the cross F1 (Linfen5064×Nongda 3338)/(Jinmai47×Jinmai84). The HMW-GS combinations differed between the four cultivars. Linfen5064 has been used extensively as a parent for high-quality wheat breeding in China. More than 80 varieties promoted in China are pedigrees from Linfen5064 (Wei et al., 2013; Li, 2010; Qiao et al., 2018). Nongda3338 is the major parent of a water-saving dwarf wheat grown in the winter wheat region of northern China, with more than 20 descendants. Jinmai47 is the dryland variety with the largest cumulative promotion area in China, with a cumulative production of approximately 200 million metric tons (Song et al., 2017). Jinmai84 has shown a good response to irrigation and fertilization in the winter wheat area of the Loess Plateau in China. Selecting these four widely used varieties as parents for research can provide information of immediate value for breeding new varieties of wheat with improved steamed bread quality.

### 2.2 Field evaluation

The DH population was planted in the Yaodu district, Shanxi province of China, at Linfen (36°08′N, 111°52′E, altitude 450 m) in 2019–2020 and 2020–2021. The seed was sown in 6 m^2^ plots, spaced 0.3 m apart and 5 m long, at 21 seeds per row. Two irrigation regimes were used each year: one irrigation (I1) at the overwintering stage and three times (I3) at the overwintering, jointing, and grain filling stages, which are treated separately. Each watering applied for each regime was 700 m^3^/hm^2^. The average rainfall was 254.2 mm in the first year and 158.7 mm in the second (Supplementary Figure S1). Standard field management practices for the region were used. After 3 months of storage, normal mature grains were used to test grain and flour characteristics. There was no climate disaster in production, and the wheat grew normally.

### 2.3 Identification of HMW-GSs

Glutenin proteins were extracted according to Singh et al. (1991) and prepared for SDS-PAGE analysis. HMW-GSs were classified following the nomenclature of Payne and Lawrence (Payne and Lawrence, 1983). From each plot, 3–5 seeds were randomly selected for the glutenin extraction, with the Chinese Spring (N, 7+8, 2+12) and Shiluan02-1 (1, 7+9, 5+10) varieties serving as standards.

### 2.4 Determination of rheological properties of dough

Grain test weight was assessed according to the Chinese National Standard for test weight measurement (GB/T5498-2013). The protein content (14% MB) was recorded using DA7200 near-infrared analysis (Perten, Sweden). Seeds of lines with the same subunit combinations were mixed in equal amounts and then milled in a cyclone mill 3100 (Perten, Sweden) (Salmanowicz et al., 2014). The Zeleny sedimentation value was measured using a Brabender shaker according to the American Association of Cereal Chemists (AACC) Method 56-63, and the result was corrected to 14% moisture content. Wet gluten content was measured with a GM2200 gluten quantity and quality measurement system. A Brabender Farinograph (AACC-54-21) and Brabender Extensograph (AACC-54-10) were used to measure the rheological properties of the dough, including dough development time, stability time tractility, stretch area, and maximum value. Three replicate samples were used in all tests.

### 2.5 Steamed bread making and sensory evaluation

Steamed bread was made according to Guo et al. (2022) with slight modifications. The dough is evenly divided into three pieces of equal quality and put into a 100 g steamed bread model with a diameter of 6.5 cm and a height of 3.5 cm. A trained tasting group of 10 people evaluated each steamed bread sample, including five men and five women.

### 2.6 Measurement of mass and volume

Each steamed bread sample was weighted, and the volume was measured with an automatic food volume tester BVM6640 (Perten, Sweden).

### 2.7 C-Cell image analysis

The crumb structure characteristics of steamed bread samples were analyzed using a C-Cell monochrome imaging system (CC.300, AACC-10-18.01) (Calibre Control International, United Kingdom). The C-Cell imaging system produced 48 numerical results. Based on the previous studies by Zhang et al. (2019) and Tozatti et al. (2020), only the trait parameters of the number of cells, number of holes, area of cells, cell diameter, cell volume, and cell wall thickness were used in the analysis.

### 2.8 Steamed breadcrumb texture analysis

The analysis of steamed bread texture followed the method of Zhang et al. (2019) with slight modifications. Three slices from the middle of each steamed bread sample were excised, cut into squares of uniform size, and placed in a texture analyzer for texture profile analysis.

### 2.9 Scanning electron microscopy (SEM)

Steamed bread microstructure was observed with scanning electron microscopy as described by Sun et al. (2022). Freeze-dried samples were sprayed with gold and placed under an electron microscope (ZEISS sigma 300), and the internal structure was observed.

### 2.10 Statistical analysis of data

The correlation coefficients of traits and analysis of variance were determined using SAS statistical software (SAS Institute Inc., Cary, NC, United States). All data were expressed as the mean of three replicates.

## 3 Results

### 3.1 HMW-GS genotype characterization

The HMW-GSs present in the parents and each line from the DH population were identified (Supplementary Figure S2). Sixteen HMW-GS combinations were found in the DH lines: (1, 7+8, 5+12), (1, 7+8, 2+12), (1, 6+8, 5+12), (1, 6+8, 2+12), (N, 7+8, 5+12), (N, 7+8, 2+12), (N, 6+8, 2+12), (N, 6+8, 5+12), (1, 7+9, 5+10), (1, 7+9, 5+12), (N, 7+9, 2+12), (1, 7+9, 2+12), (N, 7+9, 5+10), (N, 7+9, 5+12), and recombinant (N, 7+9, 2+10), (1, 7+9, 2+10). After removing the combinations with a small number of lines (<20 lines), there were 12 HMW-GS combinations to study (Table 1). The interference caused by other intra-population genetic backgrounds, such as LMW-GSs and gliadin, can be eliminated after mixing.

**TABLE 1:** Occurrence of HMW-GSs in the DHs.

	Glu-A1	Glu-B1	Glu-D1	Number of DHs	Ratio (%)
Linfen5064×Nongda3338	1	7+8	5+12	28	13.46
	1	7+8	2+12	11	5.42
	1	6+8	5+12	24	11.82
	1	6+8	2+12	9	4.43
	N	7+8	5+12	22	10.84
	N	7+8	2+12	65	32.02
	N	6+8	2+12	23	11.33
	N	6+8	5+12	21	10.34
Jinmai47×Jinmai84	1	7+9	5+10	50	18.59
	1	7+9	5+12	49	18.22
	1	7+9	2+10	10	3.72
	1	7+9	2+12	13	4.83
	N	7+9	2+10	25	9.29
	N	7+9	2+12	60	22.30
	N	7+9	5+10	31	11.52
	N	7+9	5+12	31	11.52

### 3.2 Quality effects of Glu-1 loci

The contribution of *Glu-A1*, *Glu-B1*, and *Glu-D1* loci on the quality of steamed bread were compared (Supplementary Table S1). For volume, glueyness, chewiness, and hardness indices, the loci contribution effects were *Glu-D1* > *Glu-B1* > *Glu-A1*. For elasticity, aspect ratio, surface color, surface structure, internal structure, and total sensory score, the loci contribution effects were *Glu-B1* > *Glu-D1* > *Glu-A1.* For specific volumes of steamed bread, the contribution effects were influenced by the wheat production environment, with *Glu-B1* > *Glu-D1* > *Glu-A1* under the I1 condition and *Glu-A1* > *Glu-B1* > *Glu-D1* under the I3 condition. Similarly, the contribution effects for the adhesion index and the sensory scores for elasticity, toughness, and viscosity were affected by the environment with *Glu-B1* > *Glu-D1* > *Glu-A1* under the one-time irrigation condition and *Glu-D1* > *Glu-B1* > *Glu-A1* under the three-time irrigation condition. The cohesiveness of steamed bread was affected by the water regimes during crop development, with *Glu-D1 > Glu-B1 > Glu-A1* under the one-time irrigation condition and *Glu-B1 > Glu-D1 > Glu-A1* under the three-time irrigation condition. In conclusion, the *Glu-D1* locus should be a focus when breeding special steamed bread varieties.

### 3.3 Subunit effects on the characteristics of steamed bread quality

#### 3.3.1 Volume parameters

Volume is a key steamed bread quality trait. Comparison of the *Glu-A1* sites 1 and Null showed that volume, aspect ratio, and specific volume of steamed bread made from lines carrying subunit 1 were larger than those carrying the subunit Null (Table 2). There were no significant subunit effects of the *Glu-B1* locus for volume; however, the specific volume of steamed bread with 6+8 was significantly higher than that of 7+9. The main difference among the subunits of the *Glu-D1* locus was in volume. The volume of steamed bread of 2+10, 2+12, and 5+10 was significantly higher than that of 5+12 under the one-time irrigation regime, with 2+12 having the largest aspect ratio effect. On the other hand, the volume of steamed bread of 2+10 was significantly higher than that of 5+12 and 5+10 (203.40 cm^3^ vs. 187.43 cm^3^ and 176.00 cm^3^) under the I3 irrigation. Furthermore, the volume of steamed bread with subunit 5+10 was greatly reduced when the crop was grown under the three-time versus the one-time irrigation schemes.

**TABLE 2:** Effects of glutenin subunits on quality traits of steamed bread.

Trait	Treatment	Glu-A1		Glu-B1			Glu-D1			
		1	Null	6+8	7+8	7+9	2+10	2+12	5+10	5+12
Volume	I1	186.85	183.98	186.67	185.07	184.00	189.80 A	185.07 A	190.80 A	148.81 B
	I3	187.60	184.63	181.47	188.47	186.27	203.40 A	182.47 BC	176.00 C	187.43 B
Aspect ratio	I1	1.38	1.41	1.44 A	1.41 AB	1.37 B	1.34 C	1.46 A	1.37 BC	1.39 B
	I3	1.46 A	1.42 B	1.44	1.44	1.43	1.44	1.41	1.47	1.43
Specific volume	I1	1.92	1.89	1.96 A	1.90 B	1.86 B	1.88	1.92	1.91	1.88
	I3	2.01 A	1.89 B	1.99 A	1.99 A	1.87 B	1.91 ab	1.91 ab	1.84 b	1.97 a
Adhesion (mJ)	I1	0.13	0.15	0.12 B	0.10 C	0.18 A	0.22 A	0.11 B	0.15 B	0.13 B
	I3	0.13	0.14	0.13	0.14	0.13	0.11 C	0.16 A	0.14 B	0.12 C
Cohesiveness (ratio)	I1	0.53	0.54	0.54	0.52	0.55	0.53	0.58	0.53	0.52
	I3	0.55	0.54	0.59 A	0.53 B	0.52 B	0.52	0.54	0.52	0.56
Elasticity (mm)	I1	6.40	6.33	6.53 B	6.59 A	6.14 C	6.23 B	6.26 AB	6.15 B	6.48 A
	I3	6.22	6.24	6.10 B	6.45 C	6.19 B	6.45	6.22	6.18	6.22
Glueyness (N)	I1	131.20	141.00	146.51	152.00	126.22	148.57 a	148.34 a	107.66 b	140.65 ab
	I3	143.50	129.11	144.48 A	142.09 A	124.53 B	100.78 B	131.88 A	144.65 A	136.86 A
Chewiness (mJ)	I1	931.28	1002.73	945.66	990.51	989.74	1181.00 a	995.76 ab	845.46 b	981.29 ab
	I3	1004.29 A	897.16 B	866.36 B	906.21 AB	979.46 A	827.33 C	876.19 BC	1133.11 A	912.06 B
Hardness (N)	I1	278.91	286.16	265.38	292.69	288.45	306.80	276.30	262.30	290.77
	I3	300.98 A	270.16 B	241.82 C	263.43 B	308.24 A	251.11 B	264.25 B	358.59 A	267.35 B
Number of cells	I1	4561.70	4354.03	4079.13 B	4535.67 A	4539.10 A	4447.40 AB	4125.40 B	4487.10 A	4546.87 A
	I3	4036.55 b	4382.75 a	3967.20 B	3998.33 B	4551.93 A	4705.60	4259.67	4161.30	4233.50
Number of holes	I1	6.19	5.39	5.78 B	8.61 A	4.12 B	2.44 C	5.48 AB	3.03 BC	7.16 A
	I3	4.59	4.77	3.62	5.09	5.06	0.66	2.57	2.10	3.04
Area of cells	I1	0.94	0.99	1.32 A	1.29 A	0.64 B	0.55 b	1.17 a	0.60 b	1.07 ab
	I3	1.04	0.92	1.27	1.30	0.84	0.48	0.90	1.12	1.22
Cell diameter	I1	0.94	0.98	1.03 A	0.94 B	0.95 B	1.00 AB	1.03 A	0.98 AB	0.93 B
	I3	1.08 a	0.99 b	1.06 B	1.13 A	0.94 C	0.93	1.00	0.96	1.06
Cell volume	I1	20.62	22.35	29.75 A	25.35 B	16.00 C	15.46 B	25.95 A	14.74 B	23.09 A
	I3	23.30	21.55	21.41 AB	27.03 A	20.05 B	15.50	21.56	20.98	23.91
Wall thickness	I1	0.34	0.34	0.36 A	0.34 B	0.33 B	0.33 B	0.35 A	0.34 B	0.34 B
	I3	0.36 A	0.34 B	0.35 B	0.37 A	0.34 C	0.33	0.34	0.34	0.35

The lowercase letter after the peer average indicates *P* < 0.05, and the uppercase letter indicates *P* < 0.01. I1: one-time irrigation regime at the overwintering stage, I3: three-time irrigation regime at the overwintering, jointing, and filling stages.

#### 3.3.2 Texture parameters

Hard-steamed bread requires greater cohesiveness, glueyness, elasticity, chewiness, and hardness and less adhesiveness, which is why its internal structure is fine and uniform, the texture is elastic, and the bite force is strong and ductile and does not stick. The subunit effects of the *Glu-A1* loci on texture parameters were like the volume effects, where glueyness, chewiness, and hardness produced by subunit 1 steamed bread were significantly higher than those of subunit Null (N). The texture parameters of subunit 1 steamed bread were less affected by irrigation during grain production. The adhesiveness of steamed bread produced from grain with subunit 7+8 at the *Glu-B1* locus was significantly less than other subunits, whereas the elasticity and glueyness were significantly greater than that of the subunit 7+9, and the cohesiveness and glueyness associated with the 6+8 subunit were greater. The chewiness and hardness associated with subunits 7+8 and 7 +9steamed bread were significantly greater than those for subunit 6+8, while the texture parameters of subunits 7+8 and 7+9 steamed bread were less affected by the irrigation regime during grain production. The steamed bread of subunit 2+10 at the *Glu-D1* loci had less adhesiveness and greater glueyness and chewiness. The adhesiveness of 5+12 steamed bread was smaller, while the cohesiveness, elasticity, adhesiveness, and chewiness were significantly greater than those of other subunits. The texture parameters of subunits 2+10 and 2+12 steamed bread were less affected by the irrigation regime. In contrast, the glueyness, chewiness, and hardness of subunit 5+10 steamed bread were significantly affected by the irrigation regime (Table 2).

#### 3.3.3 C-Cell analysis

The interior stomatal structure of steamed bread can be quantitatively analyzed using C-Cell image analysis technology to assess the size, number, and uniform distribution of the cells. In contrast with high-quality steamed bread, the internal stomata of poor-quality steamed bread are larger and unevenly distributed. The number of cells, cell diameter, and wall thickness of subunit N steamed bread at the *Glu-A1* loci were indicative of better quality than those of subunit 1 and were unaffected by the irrigation regime. The number of holes, area of cells, cell diameter, cell volume, and wall thickness of steamed bread with subunit 6+8 at the *Glu-B1* locus were significantly smaller and were less affected by the irrigation regime. The number of cells in steamed bread of subunit 7+8 was significantly higher than subunit 6+8, and the area of cells was significantly less than that of subunit 7+9. The number of cells, holes, and wall thickness were affected by the irrigation regime. The number of cells in steamed bread in subunit 7+9 steamed bread was significantly greater than that of subunit 6+8, and the number of holes in steamed bread was significantly less than that of subunit 7+8. In addition, the cell diameter was smaller, and the cell volume was less affected by the irrigation regime. The effects of the four subunits of *Glu-D1* loci on the C-cell system were different only under one irrigation condition. The numbers of cells in subunits 5+10 and 5+12 steamed bread were significantly higher than 2+12. The number of holes in the subunit 2+10 steamed bread was significantly less than in 5+12. The area of cells in subunits 2+10 and 5+10 steamed bread was significantly less than in 2+12. The cell diameter in the subunit 5+12 steamed bread was significantly less than in 2+12. The cell volume in subunits 5+10 and 2+10 steamed bread was significantly less than in subunits 2+12 and 5+12. The wall thickness of 2+10, 5+10, and 5+12 subunit steamed bread is significantly lower than that of 2+12. The number of holes and cell volume of 5+10 steamed bread were greatly affected by irrigation during crop production, as were the number of holes and cell diameter of the 5+12 steamed bread (Table 2).

#### 3.3.4 Sensory evaluation

The sensory score of the subunit 1 steamed bread at the *Glu-A1* locus was significantly higher than that for subunit N. The specific volume score of steamed bread of subunit 6+8 at the *Glu-B1* locus was significantly higher than that of subunit 7+9 steamed bread, but the specific volume, surface color, surface structure, internal structure, viscosity score, and total score of subunit 7+8 were significantly higher than those for subunit 6+8. The aspect ratio, surface structure, internal structure, elasticity, and total score of subunit 7+9 were significantly higher than those of subunit 6+8. The surface structure and total scores of subunits 2+10 and 5+10 steamed bread at the *Glu-D1* loci were significantly higher than those of subunit 2+12 (Supplementary Table S2).

### 3.4 Effects of different subunit combinations on quality

#### 3.4.1 Volume parameters

**FIGURE 1:**
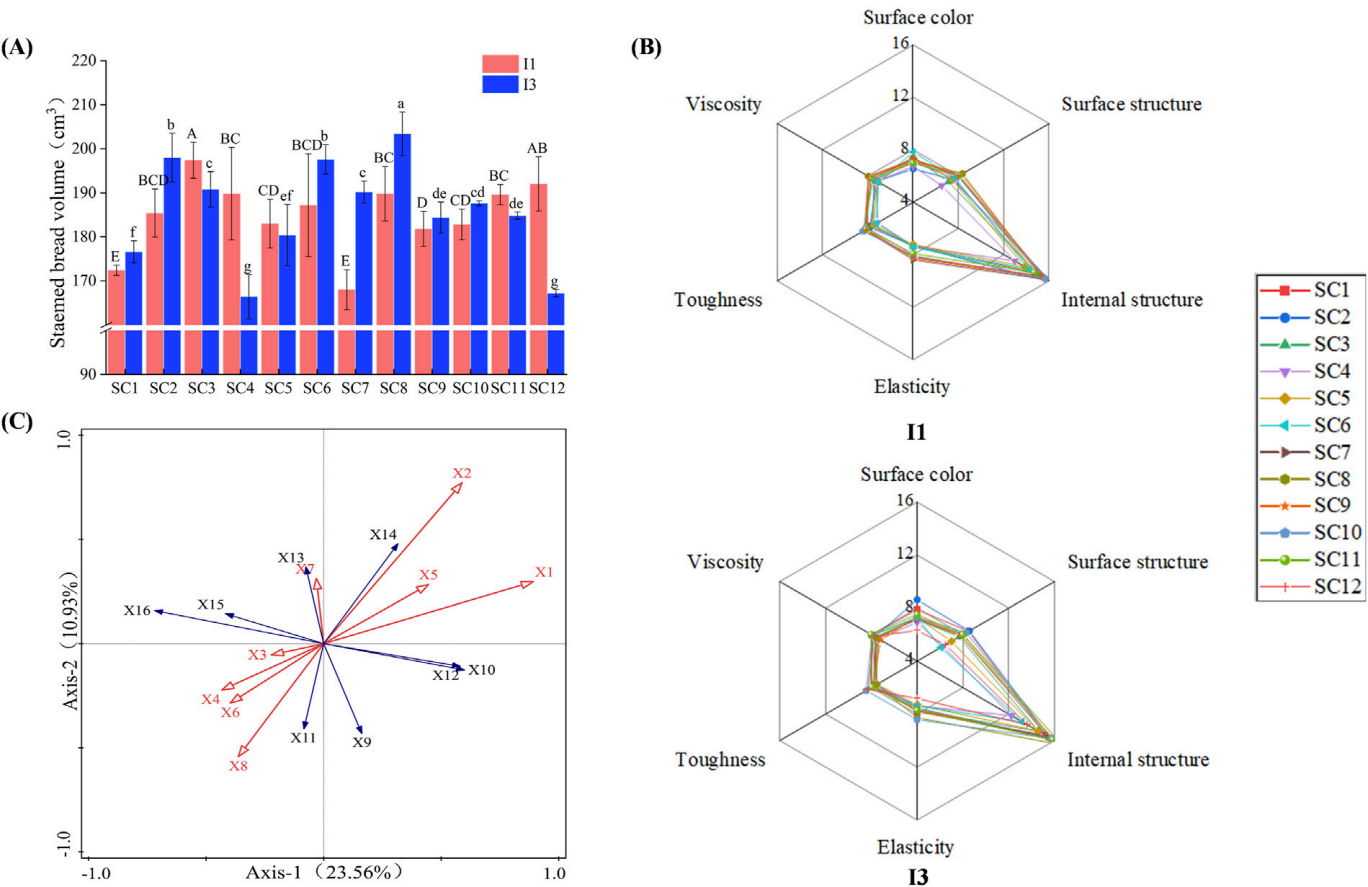
Volume, redundancy analysis, and sensory evaluation radar of different HMW-GS steamed bread flours under the I1 and I3 regimes. Uppercase letters and **, *** represent significant differences of 0.01 and 0.001 between subunit combinations, and lowercase letters and * represent significant differences of 0.05 between subunit combinations. I1: regime with one-time irrigation at the overwintering stage, I3: regime with three-time irrigation at the overwintering, jointing, and filling stages. SC1 (N, 7+8, 5+12), SC2 (1, 7+8, 5+12), SC3 (N, 7+8, 5+12), SC4 (N, 6+8, 2+12), SC5 (N, 6+8, 5+12), SC6 (1, 6+8, 5+12), SC7 (N, 7+9, 2+12), SC8 (N, 7+9, 2+10), SC9 (N, 7+9, 5+12), SC10 (1, 7+9, 5+12), SC11 (N, 7+9, 5+10), and SC12 (1, 7+9, 5+10). X1–X16, in turn, represent protein content, wet gluten content, dough development time, stability time, Zeleny sedimentation value, stretch area, tractility, maximum resistance, volume, specific volume, adhesion, cohesion, elasticity, glueyness, chewability, and hardness, respectively. **(A)**: Volume of different HMW-GS steamed bread under I1 and I3 regimes. **(B)**: Sensory evaluation radar map of different HMW-GS steamed bread under I1 and I3 regimes. **(C)**: Redundant analysis diagram of quality indexes of steamed bread and flour.

**FIGURE 2:**
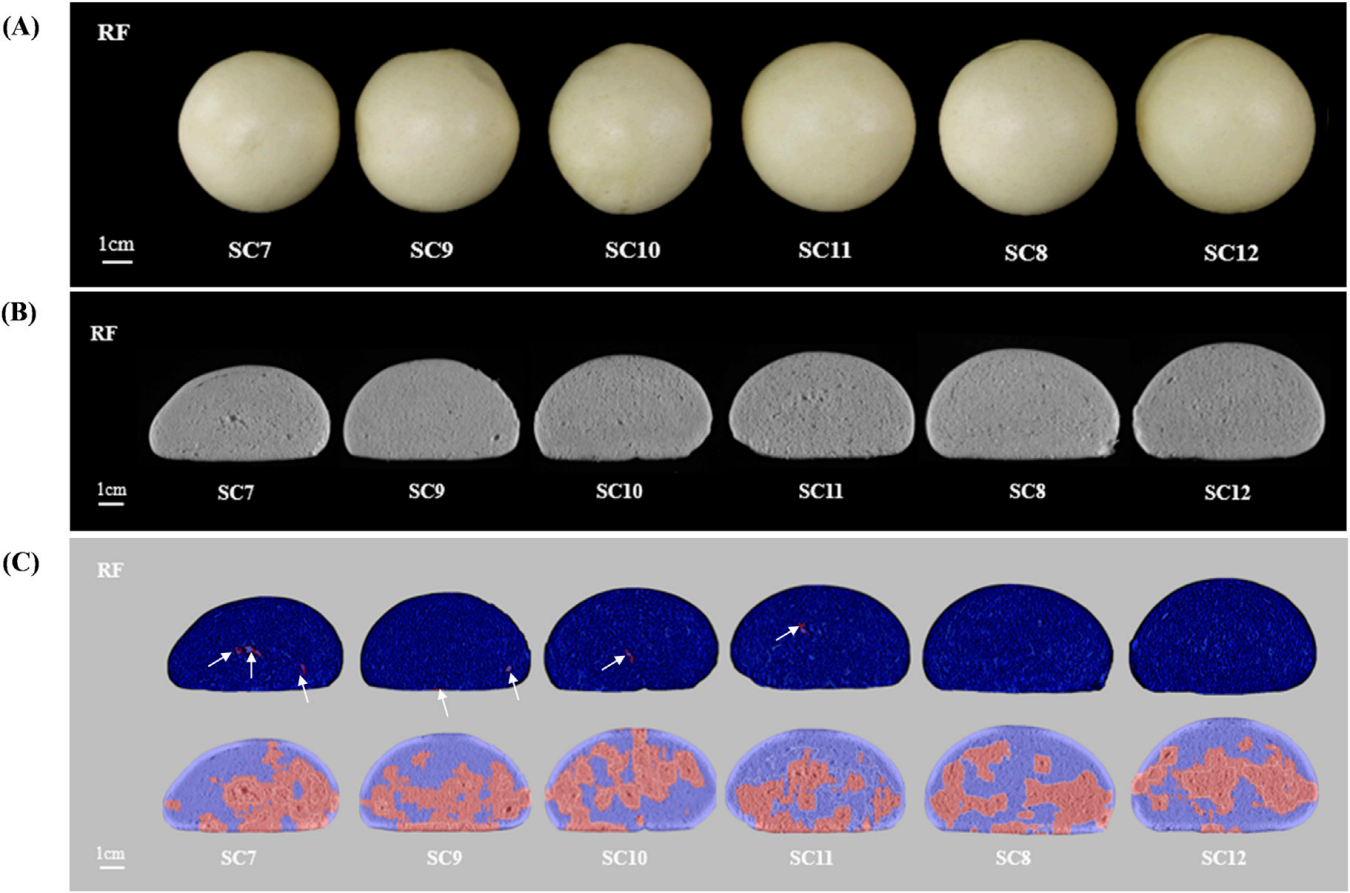
Comparison of steamed bread and cross sections of different HMW-GSs under the I1 regime. Note: **(A)** shows steamed bread made from JM47×JM84 grown under the I1 regime in order of volume from small to large; **(B)** shows a cross-section diagram of steamed bread made from JM47×JM84 grown under the I1 regime; **(C)** shows the C-Cell diagram of steamed bread made from JM47×JM84 grown under the I1 regime. The SC numbers are the same as those in Figure 1.

The differences in steamed bread volume parameters between different subunit combinations were mainly due to the subunit differences (Figures 1A, 2A). The volume and specific volume of steamed bread with subunit combinations (N, 7+8, 2+12), (1, 7+8, 5+12), and (1, 6+8, 5+12) were significantly higher than those of other combinations. The volume of (N, 7+9, 2+10) steamed bread was significantly higher than that of other combinations. The specific volume of (N, 6+8, 5+12) steamed bread was significantly higher than others. The volume of (N, 6+8, 5+12), (1, 6+8, 5+12), and (N, 7+9, 5+12) steamed bread was not affected by the irrigation regime, while the volume of (1, 7+8, 5+12), (N, 7+8, 2+12), (N, 7+9, 2+10), and (1, 7+9, 5+12) steamed bread was somewhat affected. The volume and specific volume of (N, 7+9, 2+12), (N, 7+9, 5+10), and (1, 7+9, 5+10) steamed bread were greatly affected by the irrigation regime. Thus, wheat genotypes with the combinations (N, 7+8, 2+12), (1, 7+8, 5+12), (1, 6+8, 5+12), and (N, 7+9, 2+10), which had larger volumes and were less affected by irrigation, will be valuable for breeding special steamed bread varieties.

#### 3.4.2 Texture parameters

Different subunit combinations had significant effects on texture parameters, and the combination of subunits with high-quality texture parameters always tended to be better. The hardness of steamed bread with subunit combinations (N, 7+9, 5+12) and (1, 7+9, 5+12) was significantly greater than that of other combinations. The adhesiveness of (N, 7+8, 5+12), (1, 7+8, 5+12), (N, 6+8, 5+12), and (1, 6+8, 5+12) steamed bread was significantly better than those of other combinations. The cohesiveness of (N, 6+8, 5+12) and (1, 6+8, 5+12) steamed bread was significantly higher than others, and the elasticity of (N, 7+8, 5+12), (1, 7+8, 5+12), and (N, 7+8, 2+12) steamed bread was significantly higher than that of other combinations. Steamed breads with combinations (1, 7+8, 5+12), (N, 6+8, 2+12), (N, 6+8, 5+12), and (1, 6+8, 5+12) were significantly more gluey. The chewiness of (1, 7+8, 5+12), (N, 7+8, 2+12), (N, 7+9, 5+12), (1, 7+9, 5+12), and (N, 7 +9, 5+10) steamed bread was significantly higher. The adhesiveness, elasticity, and chewiness of (N, 7+9, 2+10) steamed bread were significantly affected by the irrigation regime. The adhesiveness of (N, 7+8, 5+12), (1, 7+8, 5+12), (N, 7+9, 5+12), (N, 7+8, 2+12), and (N, 6+8, 2+12) steamed bread was greatly affected by the irrigation regime, as was the elasticity of (N, 6+8, 5+12) and (N, 7+9, 5+10) steamed bread. The glueyness of (1, 7+9, 5+10) steamed bread was affected by the irrigation regime (Supplementary Table S3).

#### 3.4.3 Differences in C-Cell images of steamed bread

The number of cells in (N, 7+9, 5+12) steamed bread was significantly higher than that of other combinations. The number of holes and area of cells in (N, 7+9, 2+10) and (N, 7+9, 5+10) steamed bread were significantly lower than those of other combinations. The stomatal diameter of (N, 7+9, 2+12), (N, 7+9, 5+12), and (1, 7+9, 5+12) steamed bread was significantly smaller than that of other combinations. The cell volume of (N, 7+9, 2+10), (1, 7+9, 5+12), and (N, 7+9, 5+10) steamed bread was significantly smaller than that of other combinations. The wall thickness of (N, 6+8, 5+12), (1, 6+8, 5+12), and (N, 7+9, 5+12) steamed bread was significantly smaller than that of other combinations (Figures 2B, C). The number of cells and holes, the cell diameter, and the wall thickness of (1, 7+8, 5+12) steamed bread were greatly affected by the irrigation regime, as was the cell diameter of (N, 7 + 8, 2 + 12) steamed bread. The irrigation regime greatly affected the cell diameter and wall thickness of (N, 6 + 8, 2 + 12) steamed bread and the number of cells in (1, 7 + 9, 5 + 12) and (1, 7 + 9, 5 + 10) (Supplementary Table S3).

#### 3.4.4 Sensory evaluation

Subunits with higher sensory scores tended to have higher steamed bread scores, such as subunits 1, 7 + 8, 7 + 9, 2 + 10, and 5 + 12. Steamed bread under the one-time irrigation regime produced significantly higher surface structure for (N, 7 + 9, 2 + 10), elasticity for (N, 7 + 9, 2 + 12), and total sensory scores for (1, 7 + 8, 5 + 12), (N, 7 + 9, 2 + 10), and (1, 7 + 9, 5 + 10). Under the three-time irrigation regime, significantly higher scores were observed for the surface color and surface structure of (1, 7 + 8, 5 + 12), internal structure and total score of (N, 7 + 9, 2 + 10), and total scores of (N, 7 + 9, 2 + 12), (1, 7 + 9, 5 + 12), and (N, 7 + 9, 5 + 10) steamed bread (Figure 1B).

The subunit combination (1, 7 + 8, 5 + 12) had greater volume, specific volume, elasticity, glueyness, chewiness, cell number, and total scores and less adhesiveness and wall thickness, fewer holes, and smaller cell diameter. The steamed bread from combination (N, 7 + 8, 5 + 12) had less adhesiveness and greater elasticity. The (N, 7 + 8, 2 + 12) steamed bread had larger volume, specific volume, elasticity, and chewiness. In contrast, the combination (N, 7 + 9, 2 + 10) had larger volume and total sensory scores and fewer holes, and (1, 7 + 9, 5 + 12) steamed bread had greater hardness and chewiness and smaller cell diameter and volume.

### 3.5 Microstructure analysis

**FIGURE 3:**
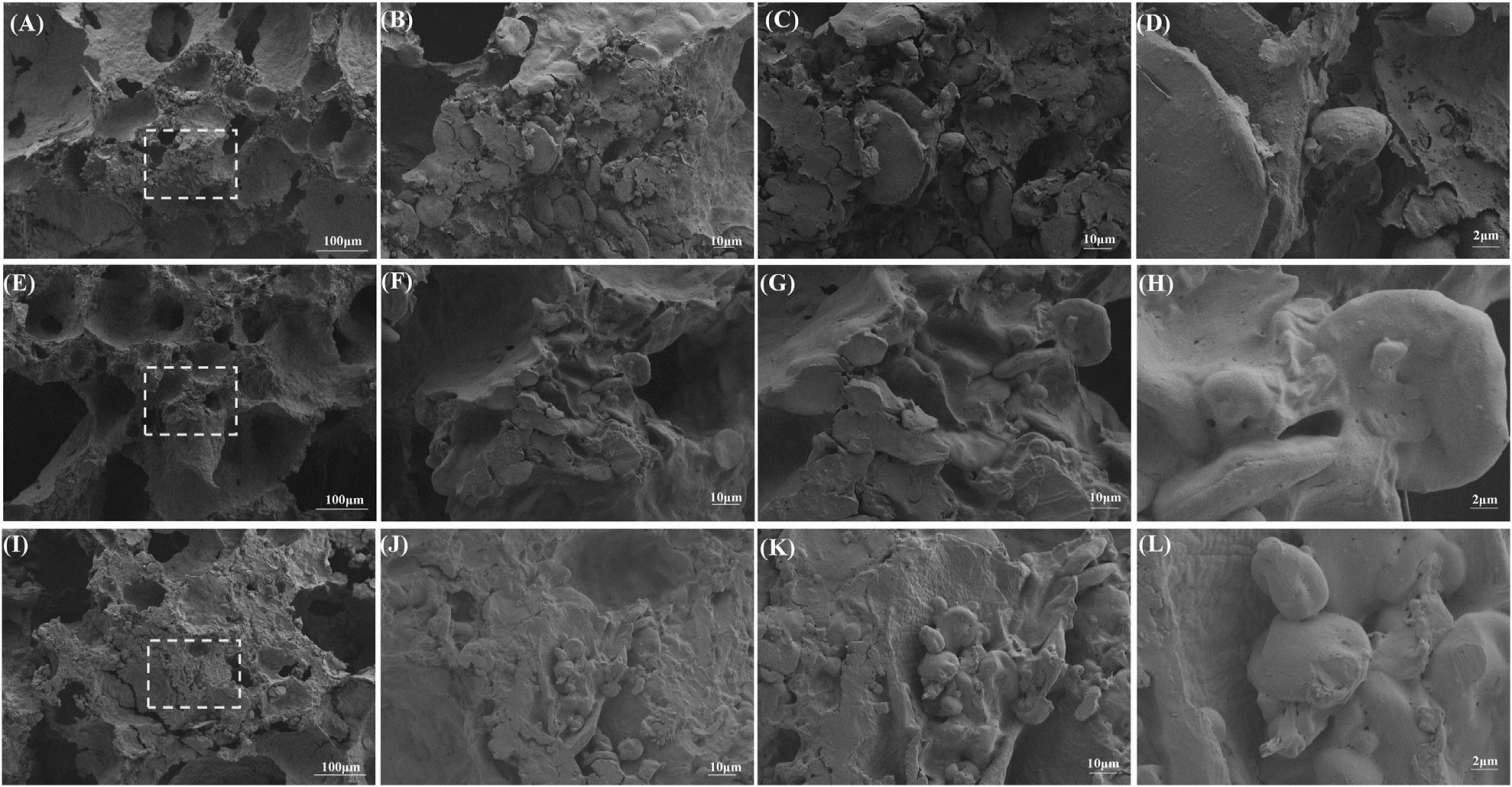
SEM images of steamed bread. From left to right, respectively, ×100, ×500, ×1000, and ×3000, **(A–D)** SEM of (1, 7+8, 5+12) steamed bread, **(E–H)** SEM of (N, 7+9, 2+10) steamed bread, and **(I–L)** SEM of steamed bread (1, 7+9, 5+12).

The steamed bread samples under the one-time irrigation regime were observed by scanning electron microscopy (SEM) (Figure 3). The internal texture was relatively uniform, with clear differences in stomatal morphology among different subunits. According to the C-Cell results, (1, 7+8, 5+12) steamed bread had more uniform and dense cells with a smooth and delicate internal cross section, while (1, 7+8, 5+12) and (N, 7+9, 2+10) had thinner walls (0.32 mm and 0.33 mm). Steamed breads (1, 7+8, 5+12) and (1, 7+9, 5+12) had smaller stomatal diameters. These differences were primarily due to the formation of the gluten network structure and the degree of filling by starch particles. SEM result shows that the starch particles are round or oval, and the gluten protein forms either flakes or filaments. The continuous structure of steamed bread was clearly observed, and the starch particles were tightly wrapped in the gluten protein network structure, which constituted the gluten network structure of the complex system together.

### 3.6 The main factors affecting steamed bread quality

There was a significant positive correlation between the volume and the stability time of steamed bread. The specific volume and cohesiveness of steamed bread were positively correlated with protein content, and glueyness was positively correlated with protein content and wet gluten content. Hardness and protein content had a significant negative correlation (Supplementary Figure S3).

The quality of steamed bread explained 23.56% and 10.93% of the flour quality index variables in the first and second axes, respectively (Supplementary Table S4). The explanation of 16 indicators in axis 1 and axis 2 are 23.56% and 10.93%, respectively. These values reflect the relationship between the quality of steamed bread and the flour quality, with certain positive and negative correlations (Figure 1C). The quality of flour affects the quality of steamed bread. The impact of each flour quality index on the quality of steamed bread was, in descending order, protein content, wet gluten content, and stability time. The contribution of protein content to the quality of steamed bread was 50.7%. The results showed that the protein content was the most critical flour quality factor affecting steamed bread quality.

### 3.7 Effect of subunits on flour quality

The correlation analysis results between the quality of steamed bread and flour showed that the protein content, wet gluten content, and stability time were the main factors affecting the volume, specific volume, cohesion, glueyness, and hardness. Steamed bread with subunits 6+8 and 5+12 had the highest protein content and better cohesiveness because of the highest protein content and better cohesiveness of the 6+8 and 5+12 subunits. Because the protein and wet gluten content of 6+8 was significantly higher, the wet gluten content of 7+8 and the protein content of 2+12, 5+10, and 5+12 were found to be higher, resulting in a better adhesiveness of their steamed bread. Because the protein content of 7+9 and 5+10 was relatively small, the steamed bread was harder, and because the dough stability time of the 2+10 and 5+10 subunits was significantly longer, the volume of steamed bread was larger. Moreover, the 2+12 and 5+12 subunits were significantly higher in terms of the protein and wet content of the flour, thus enhancing glueyness.

Combined with the quality of steamed bread, the effects of different subunits on flour quality were further analyzed (Supplementary Table S5). Regarding the stability time of the *Glu-A1* locus, subunit 1 was significantly higher than that of N only under the three-time irrigation regime. In terms of the content of wet gluten at the *Glu-B1* locus, the subunits 6+8 and 7+8 were significantly higher than that of 7+9, and the stability time of 7+8 and 7+9 was significantly higher than 6+8. The protein content and stability time of 2+12 at the *Glu-D1* locus were significantly higher, but the protein content of 5+12 was affected by the irrigation regime and was higher only under the one-time irrigation regime. The wet gluten content of 2+12 and 5+12 was significantly higher than that of 2+10 only under the one-time irrigation regime.

### 3.8 Effect of HMW-GSs on flour quality

There were differences in the quality of flour with different subunits, which led to differences in the quality of flour in different combinations and finally affected the quality of steamed bread. Significant longer stability time of the (N, 7+8, 2+12), (1, 7+8, 5+12), and (N, 7+9, 2+10) combinations increased the bread volume. Due to the high protein content, subunits (N, 6+8, 5+12) and (1, 6+8, 5+12) had larger specific volume and cohesiveness. The high protein content of (N, 6+8, 2+12), (N, 6+8, 5+12), and (1, 6+8, 5+12) and the lower wet gluten content of (N, 6+8, 5+12) and (1, 7+8, 5+12) resulted in greater glueyness of the final product. In addition, the significantly lower protein content of combinations (N, 7+9, 5+12) and (1, 7+9, 5+12) produced harder steamed bread.

Combined with the quality of steamed bread, the flour quality of different subunit combinations was analyzed further (Table 3). The subunit combination (N, 7+9, 2+10) had the longest stability time and the least protein. Conversely, the (N, 6+8, 2+12), (N, 6+8, 5+12), and (1, 6+8, 5+12) combinations had more protein, while (N, 7+9, 5+12), (1, 7+9, 5+12), and (N, 7+9, 5+10) had less protein and were greatly influenced by the irrigation regime. The wet gluten content of (N, 7+8, 5+12), (N, 7+8, 2+12), and (N, 6+8, 2+12) was significantly higher than other combinations, and (N, 7+8, 2+12), (1, 7+8, 5+12), and (N, 7+9, 2+10) had longer stability time.

**TABLE 3:** Effects of different HMW-GSs on flour quality traits.

Traits	Treatment	(N, 7+8, 5+12)	(1, 7+8, 5+12)	(N, 7+8, 2+12)	(N, 6+8, 2+12)	(N, 6+8, 5+12)	(1, 6+8, 5+12)	(N, 7+9, 2+12)	(N, 7+9, 2+10)	(N, 7+9, 5+12)	(1, 7+9, 5+12)	(N, 7+9, 5+10)	(1, 7+9, 5+10)
Test weight (g/L)	I1	732.00 ± 1.41 B	736.00 ± 0.00 A	729.00 ± 1.41 B	721.00 ± 1.41 D	724.00 ± 1.41 CD	711.00 ± 1.41 E	722.00 ± 1.41 CD	721.00 ± 0.00 D	732.00 ± 4.24 B	729.00 ± 2.83 B	731.00 ± 1.41 B	725.00 ± 1.41 C
	I3	784.00 ± 5.66 AB	781.00 ± 2.83 B	789.00 ± 1.41 A	787.00 ± 0.00 A	784.00 ± 1.41 AB	770.00 ± 0.00 C	726.00 ± 1.41 G	733.00 ± 1.41 B	742.00 ± 1.41 D	727.00 ± 0.00 FG	729.00 ± 5.66 EFG	732.00 ± 2.83 EF
Protein content (%)	I1	13.05 ± 0.27 B	12.28 ± 0.08 C	13.25 ± 0.35 B	13.99 ± 0.03 A	12.99 ± 0.03 B	13.25 ± 0.35 B	13.65 ± 0.31 A	12.59 ± 0.06 CD	12.34 ± 0.18 CD	12.80 ± 0.04 BC	12.16 ± 0.13 D	13.15 ± 0.30 B
	I3	14.14 ± 0.06 C	14.04 ± 0.06 C	14.05 ± 0.03 C	14.64 ± 0.08 B	14.54 ± 0.11 B	16.36 ± 0.17 A	11.96 ± 0.03 DE	11.06 ± 0.04 G	11.32 ± 0.11 F	11.83 ± 0.03 E	10.92 ± 0.11 G	12.08 ± 0.01 D
Wet gluten content (%)	I1	43.00 ± 1.51 A	39.20 ± 2.78 BC	41.73 ± 0.59 AB	41.77 ± 0.95 AB	38.67 ± 2.70 BCD	39.17 ± 3.86 BC	38.87 ± 1.76 BCD	34.60 ± 0.92 E	36.53 ± 1.18 CDE	37.43 ± 2.54 CDE	35.50 ± 0.62 DE	37.20 ± 0.95 CDE
	I3	42.40 ± 0.53 AB	38.40 ± 0.56 ABCD	40.20 ± 1.40 ABC	42.20 ± 4.01 AB	37.57 ± 1.19 BCD	43.70 ± 3.11 A	34.37 ± 3.32 CD	34.37 ± 2.48 CD	34.57 ± 2.68 CD	34.97 ± 0.50 CD	33.40 ± 0.52 D	38.67 ± 8.56 ABCD
Dough development time (min)	I1	2.60 ± 0.2 bc	3.00 ± 0.2 abc	2.90 ± 0.1 abc	2.50 ± 0.3 c	2.60 ± 0.2 bc	2.70 ± 0.2 abc	2.90 ± 0.5 abc	3.20 ± 0.4 a	2.50 ± 0.2 bc	3.00 ± 0.3 ab	3.00 ± 0.4 ab	3.00 ± 0.2 ab
	I3	2.80± 0.2 AB	3.20 ± 0 A	3.00 ± 0.1 AB	2.70 ± 0.3 AB	2.60 ± 0.2 B	2.90 ± 0.3 AB	2.50 ± 0.2 B	3.10 ± 0.3 A	2.50 ± 0.3 B	2.60 ± 0.3 B	3.10 ± 0.3 A	3.10 ± 0.3 A
Dough stability time (min)	I1	3.30 ± 0.2 CD	4.00 ± 0.2 AB	3.70 ± 0.4 BC	2.40 ± 0.3 F	2.60 ± 0.1 EF	2.70 ± 0.1 EF	2.90 ± 0.2 DE	4.40 ± 0.5 A	3.10 ± 0.2 DE	3.90 ± 0.4 B	4.10 ± 0.1 AB	3.30 ± 0.4 CD
	I3	3.00 ± 0.4 FG	4.10 ± 0.2 BC	3.70 ± 0.3 CD	2.50 ± 0.1 H	2.50 ± 0.2 H	3.20 ± 0.4 EF	2.70 ± 0.1 GH	4.60 ± 0.5 A	3.00 ± 0.2 FG	3.30 ± 0.2 DEF	4.50 ± 0.2 AB	3.50 ± 0.4 DE
Stretch area (cm^2^)	I1	48.50 ± 5.80 CD	61.00 ± 6.06 B	51.00 ± 2.94 C	27.75 ± 1.71 F	45.25 ± 1.26 D	39.25 ± 4.11 E	48.25 ± 3.77 CD	71.50 ± 2.89 A	57.50 ± 6.95 B	61.25 ± 4.5 B	59.75 ± 2.50 B	57.25 ± 4.50 B
	I3	50.25 ± 2.50 BC	63.25 ± 10.28 A	56.25 ± 5.74 B	34.25 ± 3.10 E	35.50 ± 4.43 E	45.25 ± 3.40 CD	40.25 ± 4.27 DE	63.50 ± 5.74 A	45.50 ± 2.08 CD	51.75 ± 4.19 BC	50.75 ± 5.38 BC	46.75 ± 4.03 CD
Maximum resistance (EU)	I1	222.25 ± 23.36 B	259.75 ± 7.27 A	225.75 ± 20.37 B	195.75 ± 11.53 C	256.00 ± 12.11 A	228.50 ± 11.82 B	226.25 ± 6.40 D	336.25 ± 12.01 A	271.00 ± 6.78 C	286.00 ± 26.98 C	314.25 ± 15.69 B	276.00 ± 18.29 C
	I3	216.50 ± 7.33 DE	259.25 ± 10.50 B	239.75 ± 14.64 C	198.50 ± 14.39 F	207.50 ± 13.96 EF	222.50 ± 6.03 CDE	205.50 ± 23.56 EF	328.75 ± 16.96 A	227.50 ± 5.45 CD	211.75 ± 16.03 DEF	267.75 ± 11.93 B	235.50 ± 6.86 C
Tractility (mm)	I1	151.50 ± 5.26 AB	158.25 ± 13.40 A	157.25 ± 23.50 A	117.25 ± 9.81 E	121.75 ± 6.85 E	122.25 ± 11.44 DE	144.50 ± 6.25 AB	143.50 ± 11.82 AB	139.00 ± 12.11 BC	141.25 ± 4.57 BC	127.25 ± 3.59 CDE	137.00 ± 3.65 BCD
	I3	154.50 ± 3.51 B	171.00 ± 3.74 A	152.25 ± 8.42 BC	126.50 ± 3.42 F	124.00 ± 11.20 F	142.50 ± 10.66 CD	138.50 ± 10.47 DE	126.00 ± 6.68 F	134.25 ± 6.18 DEF	160.50 ± 10.34 AB	127.00 ± 11.28 F	130.00 ± 8.37 EF
Zeleny sedimentation value/mL	I1	24.57 ± 0.35 BC	25.36 ± 0.90 B	27.60 ± 0.66 A	23.20 ± 0.26 E	24.34 ± 0.12 CD	23.06 ± .06 E	25.28 ± 0.36 B	23.42 ± 1.13 E	23.63 ± 0.23 DE	24.35 ± 0.38 CD	25.28 ± 0.33 B	24.30 ± 0.36 CD
	I3	25.84 ± 0.40 B	27.16 ± 0.14 A	26.14 ± 0.09 B	21.46 ± 0.27 G	22.93 ± 0.22 D	24.46 ± 0.06 C	20.50 ± 0.46 I	22.43 ± 0.25 E	19.93 ± 0.31 J	21.87 ± 0.15 F	20.90 ± 0.10 H	21.13 ± 0.06 GH

The lowercase letter after the peer average indicates *P* < 0.05, and the uppercase letter indicates *P* < 0.01. I1: one-time irrigation regime at the overwintering stage, I3: three-time irrigation regime at the overwintering, jointing, and filling stages.

## 4 Discussion

### 4.1 Ideal materials for evaluating the effects of HMW-GSs on steamed bread quality

Selecting appropriate HMW-GSs can enhance the breeding of special varieties for steamed bread production, but only a few reports have described the relationship between subunit constitution and steamed bread quality. Deng et al. (2007) used three varieties and showed that subunits 1 and 2 were positively correlated with the volume of steamed bread, and subunits 5+10 were positively correlated with the elasticity of steamed bread. Zhang et al. (2014) used 16 varieties and found that the quality of steamed bread with the presence of subunits 2* and 2+12 produced better quality. Using 33 varieties, Zhang and Li (1993) found that subunit 5+10 increased steamed bread volume. Some previous results showed inconsistencies, which were possibly related to the small number of genotypes used in the experiments and interference by differences in genetic background.

Integration of NILs, RILs, or DHs with the same HMW-GSs provides better material for evaluating the quality of steamed bread (Nieto-Taladriz et al., 1994; Popineau et al., 1994; Zhang et al., 2014; Takata et al., 2000). Using NILs, Zhu et al. (2001) demonstrated that the basis for the quality difference between 5 + 10 and 2+12 steamed bread was that 5+10 had a higher protein level. Langner et al. (2017) found in a study with DHs that subunits 7+8 and 5+10 benefited dough rheological properties. However, constructing such populations from parental hybridization to background homozygosity is a time-consuming process. In addition, it takes a long time to construct such a population from parental hybridization to background homozygous, and the workload of planting and harvesting is large, resulting in fewer subunits and combination types that can be accurately evaluated. In addition, although it is known that subunits 13+16 and 5' + 12 and other subunits affect dough rheological characteristics, there are few studies on the effects of these subunits on the quality of steamed bread. In the existing studies, a few varieties were planted in the same year and in the same place, but in the present study, the quality traits of nine subunits were investigated for 2 years with good repeatability. Among DH parents, Linfen5064 is a major high-quality wheat parent in China, from which more than 80 varieties have been derived (Wei et al., .2013; Li, 2010; Qiao et al., 2018). Nongda3338 is one of the primary parental genotypes used to develop winter wheats in northern China, with more than 20 derived varieties. Jinmai47 is a dryland variety with a cumulative area of approximately 200 million acres in China (Song et al., 2017), and Jinmai84 is one of the best winter wheat varieties for the Loess Plateau in China. Generating a research population from these materials, whose value has been verified through use in breeding and practical agriculture, provides information of immediate benefit for breeding improved steamed bread varieties.

### 4.2 Effect of gluten subunits on the quality of steamed bread

Recently, the effect of HMW-GSs on the quality of wheat flour products has been emphasized, but studies have mainly concentrated on baked bread quality. Payne (1987) reported that HMW-GS composition could determine about 67% of the genetic variation in bread quality. Zhao et al. (2020) demonstrated that the relative bread volume and score for subunits at *Glu-A1* were 2* > 1 > N, and those for *Glu-B1* were 14+15 > 13+16. They also found that the best volume and taste scores were produced in bread with subunit 5+10 at the *Glu-D1* locus. Few reports describe the effects of different subunit variation types of *Glu-1* on steamed bread. Zhang et al. (2014) suggested that subunit 7+8 was good for making steamed bread, and 5+10 steamed bread has better quality than 2+12. This result differs from the present study, possibly because fewer varieties were used. The present research studied nine subunits and their effects on 24 indices, including volume traits, physical properties, and sensory scores of steamed breads. Subunit 1 of *Glu-A1*, subunits 7+8 and 7 +9of *Glu-B1*, and subunits 5+12 and 2+10 of *Glu-D1* enhanced the quality of steamed bread, and their volume traits and physical properties were less affected by the irrigation regime used during crop production.

Not all the effects of the best subunits were optimal. The physical properties such as chewiness, hardness, and number of cells, and sensory scores from subunits 7+8 and 7+9 at the *Glu-B1* locus were significantly better than those of subunit 6+8 steamed bread, but the number of holes, area of cells, and cell diameter were not. The steamed bread volume, physical properties such as chewiness and area of cells, and sensory scores of subunits 2 + 10 and 5+12 at the *Glu-D1* locus were better than those of subunit 2+12 steamed bread, but the aspect ratio and glueyness of steamed bread of subunit 2+12 were larger. Therefore, there were differences in the volume traits, physical properties, and sensory scores of steamed breads with different subunit combinations. The recombinant subunit 2+10 obtained in the present work may have great value for developing improved wheat cultivars for the steamed bread market. In the subsequent selection of cultivars suitable for producing high-quality steamed bread, the varieties containing these subunits should be screened according to the actual conditions and subjected to different growing regimens.

### 4.3 Flour quality is the main determinant of steamed bread quality

After improving the mixing properties and water absorption of dough, different types of flour can be produced, targeted for end use as bread, steamed bread, and other products (Kumar et al., 2019). Most of the dough for making bread has a stabilization time of more than 8 min, protein content of more than 14%, and wet gluten content of more than 30%, whereas steamed bread requires a dough stability time of 3–5 min, protein content of more than 12.5%, and a wet gluten content between 28% and 33% (Chinese National Standard GB/T 17320-2013). Thus, although wheat cultivars may contain the same subunit, the flour produced must meet requirements for making different end products. It is generally considered that the dough rheological properties of the subunit 7+8 at the *Glu-B1* site are better than those of the subunit 7+9, and the subunit 5+10 at the *Glu-D1* site has the best dough rheological properties. Hence, the alleles containing 7+8 at *Glu-B1* and 5+10 at *Glu-D1* exhibited better rheological properties than other combinations. Current research on wheat quality mainly focuses on the analysis of processing quality, while research on the interaction between processing quality and steamed bread quality is only beginning. Li et al. (2001) showed that protein content and wet gluten content were the main factors causing differences in steamed bread quality. Consistent with the results of the present study, Zhao et al. (2018) demonstrated that stability time has a significant effect on the shape and internal structure of steamed bread. We found that protein content was the most critical factor responsible for differences in steamed bread quality, followed by wet gluten content and stability time. Future breeding research should assess if varying the combination of *Glu-1* loci will enhance steamed bread quality.

### 4.4 Influence of irrigation regimes on the effects of HMW-GSs on quality

Appropriate moisture during the growth process of wheat will improve the dough development and stabilization time of the resulting crop, but excessive irrigation can adversely affect the composition and protein content of the grain. Compared to protein content, the gluten macro polymer (GMP) content is more sensitive to the production environment. GMPs are composed of HMW-GSs and LMW-GSs connected and polymerized through disulfide bonds, and their content and properties directly affect the processing quality of flour (Don et al., 2003a; Don et al. 2003b; Don et al. 2005). Irrigation is conducive to the accumulation of glutenin in strong and medium-gluten wheat and the formation of large-size GMP particles, whereas water deficiency promotes the accumulation of gluten in weak-gluten wheat and the particle size distribution of large-size GMP particles (Zhao et al., 2011). The composition and expression level of HMW-GSs affected the formation of GMPs, which in turn affects flour quality (Weegels et al., 1995). Different HMW-GSs will generally show different accumulation forms under different environmental conditions. Furthermore, the expression and proportion of HMW-GS alleles can be altered by irrigation (Jiang et al., 2009). Some previous studies have discussed the effects of irrigation methods on the processing quality of wheat with different genetic backgrounds, but few studies have examined the effects of irrigation methods on different HMW-GSs and processing quality.

Zhao et al. (2020) and Zhou et al. (2018) found that the stability time, stretching area, and tractility of subunits 1 and 5+10 were greatly affected by moisture, a result that is consistent with the present study. We also found that the protein content, tractility, and stretching area of the subunits 6+8, 7+8, 7+9, and 2+10 were significantly affected by the irrigation regime during production. The Zeleny sedimentation value of subunits 2+12 and the maximum resistance of subunits 5+ 12were also significantly affected by irrigation. Linfen5064 contains subunits 7+8 and 5+12, whose overall dough rheological properties are less affected by water during crop growth and contribute to good cooking quality. These characteristics are the primary reasons why Linfen5064 is commonly used as a parent for breeding high-quality wheat. Therefore, appropriate irrigation conditions should be used during crop production of different HMW-GS special steamed bread varieties to ensure the highest quality end products.

## Data Availability

The original contributions presented in the study are included in the article/Supplementary Material, further inquiries can be directed to the corresponding authors.
