# Supplementary material for: Effects of high-molecular-weight glutenin subunit on hard-steamed bread quality
Source: Front Genet. 2024 Oct 3;15:1473518. doi: 10.3389/fgene.2024.1473518 (PMC11484002; doi:10.3389/fgene.2024.1473518)
Supplement: Supplementary file 1 [file Table2.DOCX]

Effects of high-molecular-weight glutenin subunit on hard-steamed bread quality

**Jing Zhao^1^**^†^**^,2^**, **Tianyi Wang^1^**^†^**^,2^**, **Hui Chen^1,2^**, **Jiajia Zhao^1^**, **Ling Qiao ^1^**, **Bangbang Wu^1^**, **Yuqiong Hao^1^**, **Chuan Ge^1^**, **Juanling Wang^2^**, **Zhiwei Feng^2*^**, **Xianghai Meng^3*^**

^1^ Institute of Wheat Research, Key Laboratory of Sustainable Dryland Agriculture (Co-construction by Ministry and Province) Ministry of Agriculture and Rural Affairs Shanxi Agricultural University, Linfen, China

^2^ College of Agriculture, Shanxi Agricultural University, Taigu, China

^3^ Dryland Farming Institute, Hebei Academy of Agriculture and Forestry Sciences

*** Correspondence:**

Zhiwei Feng and Xianghai Meng

1. mail address: zhiweifeng@126.com; mengxianghai5229@163.com

| **Supplemental Table 1** Effect of Glu-1 locus on quality traits of steamed bread |
| --- |
| \|  \| Loci \| **Glu-A1** \| \| **Glu-B1** \| \| **Glu-D1** \| \| \| --- \| --- \| --- \| --- \| --- \| --- \| --- \| --- \| \|  \| Treatment \| I1 \| I3 \| I1 \| I3 \| I1 \| I3 \| \|  \| DOF \| 1 \| 1 \| 2 \| 2 \| 3 \| 3 \| \| Volume index \| Volume(cm^3^) \| 1.99 \| 1.45 \| 1.29 \| 4.82 \| 24.50^**^ \| 33.78^***^ \| \| Specific volume \| 2.2 \| 24.29^***^ \| 21.55^**^ \| 22.30^**^ \| 2.97 \| 15.45^*^ \| \| Texture index \| Adhesion (mJ) \| 1.88 \| 3.41 \| 58.56^***^ \| 25.48^***^ \| 25.48^***^ \| 46.57^***^ \| \| Cohesiveness (Ratio) \| 0.33 \| 0.93 \| 4.17 \| 11.45^*^ \| 4.17 \| 11.45^*^ \| \| Elasticity (mm) \| 1.04 \| 0.13 \| 43.36^***^ \| 25.70^***^ \| 18.38^**^ \| 6.55 \| \| Glueyness (N) \| 1.21 \| 10.93 \| 21.07^***^ \| 10.96^*^ \| 10.96^*^ \| 27.58^***^ \| \| Chewiness (mJ) \| 1.96 \| 14.16 \| 13.15^**^ \| 11.15^*^ \| 11.15^*^ \| 47.92^***^ \| \| Hardness (N) \| 0.78 \| 9.39 \| 7.71 \| 36.99^***^ \| 10.65 \| 55.19^***^ \| \| Sensory score \| Specific volume \| 42.91^***^ \| 2.52 \| 22.46^***^ \| 2.79 \| 16.34^***^ \| 48.27^***^ \| \| Aspect ratio \| 0.48 \| 0.54 \| 98.76^***^ \| 41.11^***^ \| 13.72^**^ \| 3.12 \| \| Surface color \| 0.21 \| 0.08 \| 1.17 \| 6.46^*^ \| 0.67 \| 6.11 \| \| Surface structure \| 0.37 \| 2.36 \| 19.93^***^ \| 33.68^***^ \| 15.43^***^ \| 3.61 \| \| Internal structure \| 0.59 \| 1.02 \| 7.44^*^ \| 9.98^**^ \| 0.79 \| 5.06 \| \| Elasticity \| 0.28 \| 0.79 \| 17.78^***^ \| 2.28 \| 3.87 \| 3.23 \| \| Toughness \| 0.07 \| 1.78 \| 12.42^***^ \| 3.04 \| 3.57 \| 4.27 \| \| Stickiness \| 0.28 \| 1.84 \| 7.29^*^ \| 0.24 \| 4.46 \| 3.27 \| \| Total points \| 3.53^*^ \| 1.11 \| 34.50^***^ \| 25.10^***^ \| 9.17^*^ \| 6.11 \| |
| ^*^, ^**^, and ^***^ are significant at 0.05, 0.01, and 0.001, respectively. I1: regime once at overwintering stage, I3: irrigation three times at overwintering, jointing, and filling stages. |

| **Supplemental Table 2** Sensory evaluation of steamed bread with different subunit locus variation types |
| --- |
| \| **Traits** \| **Scoring criteria:** \| **Treatment** \| **Reference sample scores** \| **Subunits** \| \| \| \| \| \| \| \| \| --- \| --- \| --- \| --- \| --- \| --- \| --- \| --- \| --- \| --- \| --- \| --- \| \| **Glu-A1** \| \| **Glu-B1** \| \| \| **Glu-D1** \| \| \| \| \| 1 \| N \| 6+8 \| 7+8 \| 7+9 \| 2+10 \| 2+12 \| 5+10 \| 5+12 \| \| **Specific volume /(mL/g) (20 score)** \| A specific volume greater than or equal to 2.8 is worth a full score of 20; a specific volume less than or equal to 1.8 will be awarded a minimum score of 5 points; the specific volume is between 2.8~1.8, and 1.5 points will be deducted for every 0.1 decrease \| I1 \| 9.19 A \| 6.77 B \| 8.05 A \| 8.67 A \| 6.77 B \| 6.80 B \| 6.50 B \| 7.90 A \| 8.11 A \| \| I3 \| 6.73 \| 7.08 \| 7.26 \| 6.77 \| 6.95 \| 8.50 A \| 7.60 B \| 5.80 D \| 6.78 C \| \| **Aspect ratio (5 score)** \| A maximum score of 5 points is awarded if the aspect ratio is less than or equal to 1.40; a minimum score of 0 is awarded for a score greater than 1.60; between 1.40~1.60, 1 point will be deducted for every 0.05 increase \| I1 \| 3.42 \| 3.72 \| 0.00 C \| 4.60 B \| 4.90 A \| 5.00 A \| 3.31 B \| 4.90 A \| 3.11 B \| \| I3 \| 3.35 \| 3.65 \| 1.46 C \| 3.67 B \| 4.52 A \| 4.20 \| 3.39 \| 4.10 \| 3.33 \| \| **Elasticity (10 score)** \| Good elasticity of finger pressing: 8 ~ 10 points; weak rebound of finger pressing: 6 ~ 7 points; finger compression does not rebound or compresses are difficult: 4 ~ 5 points \| I1 \| 7.06 \| 7.17 \| 7.34 \| 7.04 \| 7.08 \| 7.21 \| 7.17 \| 6.94 \| 7.17 \| \| I3 \| 7.40 \| 7.32 \| 7.17 b \| 7.89 a \| 7.16 b \| 7.26 \| 7.09 \| 6.90 \| 7.64 \| \| **Surface color (10 score)** \| Good glossiness 8 ~ 10 points; slightly darker 6 ~ 7 minutes; gray 4 ~ 5 points \| I1 \| 7.76 \| 7.63 \| 7.02 C \| 7.45 B \| 8.11 A \| 8.35 A \| 7.04 C \| 8.04 AB \| 7.75 B \| \| I3 \| 7.29 \| 7.67 \| 6.44 C \| 8.24 A \| 7.78 B \| 8.00 \| 7.28 \| 7.33 \| 7.67 \| \| **Surface structure (10 score)** \| Smooth surface: 8 ~ 10 points; wrinkled, collapsed, bubbles or burns: 4 ~ 7 points \| I1 \| 15.18 \| 14.76 \| 13.70 b \| 15.32 a \| 15.28 a \| 15.33 \| 14.64 \| 14.67 \| 15.03 \| \| I3 \| 14.51 \| 15.12 \| 13.35 \| 15.16 \| 15.54 \| 16.67 \| 14.16 \| 14.78 \| 15.03 \| \| **Internal structure (20 score)** \| Stomatas fine and uniform :18 ~ 20 points; the stomatas are delicate and basically uniform, with individual bubbles :13 ~ 17 points, the edge and the epidermis are separated, and 1 point is deducted; the stomatas are basically uniform but have one of the following conditions :10 ~ 12 points, too fine, there are slightly more bubbles, the stomatas are uniform but the structure is slightly rough; uneven porosity or very rough structure :5 ~ 9 points \| I1 \| 7.65 \| 7.76 \| 7.31 B \| 7.33 B \| 8.12 A \| 8.20 \| 7.73 \| 7.90 \| 7.58 \| \| I3 \| 7.48 \| 7.71 \| 7.35 \| 7.69 \| 7.79 \| 8.27 \| 7.61 \| 7.35 \| 7.63 \| \| **Toughness (10 score)** \| Strong bite, 8 ~ 10 points; the bite is average; 6 ~ 7 points; poor bite strength, slag off when cutting or chewing dry; 4 ~ 5 points \| I1 \| 7.91 \| 7.84 \| 7.29 B \| 7.68 B \| 8.24 A \| 8.32 \| 7.66 \| 8.17 \| 7.79 \| \| I3 \| 8.00 \| 7.75 \| 7.70 \| 7.66 \| 7.98 \| 7.56 \| 7.77 \| 8.21 \| 7.79 \| \| **Viscosity (10 score)** \| Refreshing and not sticky teeth 8 ~ 10 points; slightly sticky; 6 ~ 7 points chewing is not refreshing, very sticky 4 ~ 5 points \| I1 \| 7.42 \| 7.54 \| 7.12 b \| 7.34 ab \| 7.77 a \| 7.94 \| 7.46 \| 7.84 \| 7.34 \| \| I3 \| 7.83 \| 7.59 \| 7.65 \| 7.75 \| 7.67 \| 7.43 \| 7.50 \| 7.91 \| 7.72 \| \| **Tasty (5 score)** \| The inherent aroma of normal wheat, 5 points; bland taste 4 points, peculiar smell, 2 ~ 3 points \| I1 \| 5 \| 5 \| 5 \| 5 \| 5 \| 5 \| 5 \| 5 \| 5 \| \| I3 \| 5 \| 5 \| 5 \| 5 \| 5 \| 5 \| 5 \| 5 \| 5 \| \| **Total score (100 score)** \| \| RF \| I1 \| 68.19 b \| 62.83 B \| 70.43 A \| 71.28 A \| 72.15 a \| 66.50 b \| 71.35 a \| 68.88 ab \| \| TW \| I3 \| 68.90 \| 63.37 B \| 69.83 A \| 70.39 A \| 72.89 \| 67.39 \| 67.37 \| 68.59 \| |
| Lowercase letters indicate P<0.05 and uppercase letters indicate P<0.01. I1: regime once at overwintering stage, I3: irrigation three times at overwintering, jointing, and filling stages. |

| **Supplemental Table 3** Comparison of steamed bread quality in different HMW-GS |
| --- |
| \| **Traits** \| **Treatment** \| (N,7+8,5+12) \| (1,7+8,5+12) \| (N,7+8,2+12) \| (N,6+8,2+12) \| (N,6+8,5+12) \| (1,6+8,5+12) \| (N,7+9,2+12) \| (N,7+9,2+10) \| (N,7+9,5+12) \| (1,7+9,5+12) \| (N,7+9,5+10) \| (1,7+9,5+10) \| \| --- \| --- \| --- \| --- \| --- \| --- \| --- \| --- \| --- \| --- \| --- \| --- \| --- \| --- \| \| **Volume** \| I1 \| 172.4 E \| 185.40 BCD \| 197.40 A \| 189.80 BC \| 183.00 CD \| 187.20 BCD \| 168.00 E \| 189.80 BC \| 181.80 D \| 182.80 CD \| 189.60 BC \| 192.00 AB \| \| I3 \| 176.6 F \| 198.00 B \| 190.80 C \| 166.40 G \| 180.40 EF \| 197.60 B \| 190.20 C \| 203.40 A \| 184.40 DE \| 187.60 CD \| 184.80 DE \| 167.20 G \| \| **Aspect ratio** \| I1 \| 1.38 CD \| 1.33 E \| 1.53 A \| 1.44 B \| 1.45 B \| 1.43 B \| 1.41 BC \| 1.34 DE \| 1.33 E \| 1.42 BC \| 1.41 BC \| 1.34 E \| \| I3 \| 1.36 F \| 1.53 A \| 1.43 BCD \| 1.39 CDEF \| 1.50 A \| 1.43 BC \| 1.42 BCD \| 1.44 B \| 1.38 DEF \| 1.37 EF \| 1.41 BCDE \| 1.53 A \| \| **Specific volume** \| I1 \| 1.77 FG \| 1.94 ABC \| 2.00 A \| 1.98 A \| 1.94 ABC \| 1.97 AB \| 1.76 G \| 1.88 CDE \| 1.83 EF \| 1.85 DE \| 1.91 BCD \| 1.91 BCD \| \| I3 \| 1.82 D \| 2.20 A \| 1.95 B \| 1.86 D \| 1.94 B \| 2.16 A \| 1.91 BC \| 1.91 BC \| 1.85 DE \| 1.87 CD \| 1.85 DE \| 1.83 DE \| \| **Hardness (N)** \| I1 \| 281.90 BCD \| 303.97 AB \| 292.21 ABC \| 270.87 BCD \| 259.75 CD \| 265.53 BCD \| 265.82 BCD \| 306.80 AB \| 329.89 A \| 303.60 AB \| 282.07 BCD \| 242.53 D \| \| I3 \| 266.26 DE \| 264.34 DE \| 259.69 DE \| 261.92 DE \| 238.01 FG \| 225.52 G \| 271.15 D \| 251.11 D \| 295.06 EF \| 314.93 B \| 318.04 B \| 399.14 A \| \| **Adhesion (mJ)** \| I1 \| 0.09 F \| 0.10 F \| 0.10 EF \| 0.11 DEF \| 0.12 CDE \| 0.13 CD \| 0.21 A \| 0.22 A \| 0.20 A \| 0.14 C \| 0.13 C \| 0.17 B \| \| I3 \| 0.13 CDE \| 0.14 BCD \| 0.15 BC \| 0.15 B \| 0.12 EFG \| 0.11 FG \| 0.18 A \| 0.11 FG \| 0.10 G \| 0.12 DEF \| 0.15 B \| 0.13 DE \| \| **Cohesiveness (Ratio)** \| I1 \| 0.47 \| 0.54 \| 0.54 \| 0.54 \| 0.54 \| 0.54 \| 0.66 \| 0.53 \| 0.54 \| 0.52 \| 0.53 \| 0.53 \| \| I3 \| 0.54 B \| 0.53 B \| 0.53 B \| 0.53 B \| 0.61 A \| 0.63 A \| 0.55 B \| 0.52 B \| 0.52 B \| 0.51 B \| 0.51 B \| 0.52 B \| \| **Elasticity(mm)** \| I1 \| 6.66 A \| 6.54 AB \| 6.56 AB \| 6.56 AB \| 6.54 AB \| 6.50 ABC \| 5.66 F \| 6.23 DE \| 6.30 CDE \| 6.37 BCD \| 6.11 E \| 6.19 DE \| \| I3 \| 6.47 A \| 6.45 A \| 6.44 A \| 6.42 AB \| 5.87 F \| 6.02 E \| 5.80 F \| 6.45 A \| 6.18 CD \| 6.36 AB \| 6.30 BC \| 6.06 DE \| \| **Glueyness(N)** \| I1 \| 133.57 \| 163.53 \| 158.89 \| 148.56 \| 146.08 \| 144.88 \| 137.57 \| 148.57 \| 138.05 \| 117.81 \| 116.73 \| 98.58 \| \| I3 \| 144.47 B \| 142.22 B \| 139.57 BC \| 141.37 B \| 147.55 B \| 144.53 B \| 114.69 DE \| 100.78 E \| 117.87 D \| 124.53 D \| 126.58 CD \| 162.72 A \| \| **Chewiness (mJ)** \| I1 \| 884.44 \| 1058.18 \| 1028.91 \| 964.13 \| 944.47 \| 928.38 \| 994.24 \| 1181.00 \| 1113.72 \| 958.55 \| 910.91 \| 780.01 \| \| I3 \| 923.75 CD \| 905.78 CDE \| 889.11 CDE \| 896.28 CDE \| 855.70 CDE \| 847.12 CDE \| 843.18 DE \| 827.33 E \| 929.73 BC \| 1010.31 B \| 1012.24 B \| 1253.97 A \| \| **Number of cells** \| I1 \| 4567.40 CD \| 5145.2 A \| 3894.4 G \| 3947.2 G \| 4299.8 EF \| 3990.4 G \| 4534.60 CDE \| 4447.40 DEF \| 4898.40 B \| 4380.00 DEF \| 4243.00 F \| 4731.20 BC \| \| I3 \| 4390.4 D \| 3174.40 G \| 4430.2 D \| 4049.00 EF \| 3804.80 F \| 4047.800 EF \| 4299.80 DE \| 4705.60 BC \| 4872.00 AB \| 5111.60 A \| 4510.20 CD \| 3812.40 F \| \| **Number of holes** \| I1 \| 11.49 A \| 10.94 A \| 3.41 EF \| 4.51 DE \| 7.02 BC \| 5.81 CD \| 8.51 B \| 2.44 EF \| 3.97 DEF \| 3.75 DEF \| 1.78 F \| 4.28 DE \| \| I3 \| 8.14 A \| 1.91 E \| 5.22 BCD \| 5.33 BC \| 3.13 DE \| 2.41 E \| 2.29 E \| 3.53 CDE \| 7.08 AB \| 6.96 AB \| 3.40 CDE \| 7.08 AB \| \| **Area of cells** \| I1 \| 1.52 a \| 1.21 ab \| 1.15 abc \| 1.44 a \| 1.33 ab \| 1.20 ab \| 0.91 abc \| 0.55 bc \| 0.61 bc \| 0.57 bc \| 0.40 c \| 0.79 abc \| \| I3 \| 1.93 \| 1.14 \| 0.83 \| 1.18 \| 0.97 \| 1.66 \| 0.69 \| 0.48 \| 0.82 \| 0.82 \| 0.51 \| 1.74 \| \| **Cell diameter** \| I1 \| 0.88 E \| 0.86 E \| 1.08 A \| 1.11 A \| 0.97 BC \| 1.01 B \| 0.89 DE \| 1.00 B \| 0.92 CDE \| 0.96 BC \| 1.01 B \| 0.95 BCD \| \| I3 \| 1.03 CD \| 1.35 A \| 1.01 DE \| 0.97 EF \| 1.12 B \| 1.08 BC \| 1.00 DE \| 0.93 FG \| 0.88 G \| 0.89 G \| 0.94 FG \| 0.99 DEF \| \| **Cell volume** \| I1 \| 27.88 ABC \| 24.28 BCD \| 23.90 BCDE \| 33.96 A \| 29.12 AB \| 26.16 ABC \| 20.00 CDEF \| 15.46 EF \| 16.08 DEF \| 15.00 F \| 12.44 F \| 17.04 DEF \| \| I3 \| 33.48 A \| 28.68 AB \| 18.92 BC \| 23.00 ABC \| 22.58 BC \| 18.64 BC \| 22.76 BC \| 15.50 C \| 21.38 BC \| 18.70 BC \| 14.80 C \| 27.16 AB \| \| **Wall tickness** \| I1 \| 0.34 CD \| 0.32 G \| 0.37 A \| 0.37 A \| 0.35 B \| 0.35 B \| 0.32 EFG \| 0.33 CDE \| 0.32 FG \| 0.34 CD \| 0.34 BC \| 0.33 DEF \| \| I3 \| 0.35 C \| 0.40 A \| 0.35 C \| 0.34 DE \| 0.36 B \| 0.35 C \| 0.34 CD \| 0.33 EF \| 0.32 F \| 0.33 F \| 0.34 DE \| 0.35 C \| |
| The lowercase letter after the peer average indicates P<0.05, and the uppercase letter indicates P<0.01. I1: regime once at overwintering stage, I3: irrigation three times at overwintering, jointing, and filling stages. |

| **Supplemental Table 4** Importance ranking and significance test results of dough rheological properties contribution rate |
| --- |
| \| **Dough rheological properties** \| **Importance ranking** \| **Relative contribution rate** \| **F** \| **P** \| \| --- \| --- \| --- \| --- \| --- \| \| X1 \| 1 \| 50.7 \| 5.4 \| 0.01 \| \| X2 \| 2 \| 24.1 \| 2.8 \| 0.076 \| \| X4 \| 3 \| 7 \| 0.8 \| 0.408 \| \| X3 \| 4 \| 5 \| 0.5 \| 0.57 \| \| X8 \| 5 \| 4.2 \| 0.5 \| 0.608 \| \| X6 \| 6 \| 3.9 \| 0.4 \| 0.628 \| \| X5 \| 7 \| 3.7 \| 0.4 \| 0.724 \| \| X7 \| 8 \| 1.5 \| 0.1 \| 0.908 \| |
| X1-X7 represents protein content, wet gluten content, dough development time, stability time, Zeleny sedimentation value, stretch area, tractility and maximum resistance. |

| **Supplemental Table 5** Dough rheological properties of different subunit variation types |
| --- |
| \| **Traits** \| **Treatment** \| **Glu-A1** \| \| **Glu-B1** \| \| \| **Glu-D1** \| \| \| \| \| --- \| --- \| --- \| --- \| --- \| --- \| --- \| --- \| --- \| --- \| --- \| \| 1 \| Null \| 6+8 \| 7+8 \| 7+9 \| 2+10 \| 2+12 \| 5+10 \| 5+12 \| \| **Test weight (g/L)** \| I1 \| 725.25±9.84 \| 726.50±5.02 \| 718.67±6.19 C \| 732.33±3.27 A \| 726.67±4.77 B \| 721.00±0.00 \| 724.00±4.05 \| 728.00±3.65 \| 727.33±8.69 \| \| I3 \| 752.50±25.05 \| 759.25±28.11 \| 780.33±8.14 A \| 784.67±4.63 A \| 731.50±5.92 B \| 733.00±1.41 b \| 767.33±32.04 a \| 730.50±4.04 b \| 764.67±23.34 a \| \| **Protein content (%)** \| I1 \| 12.87±0.44 \| 13.00±0.63 \| 13.41±0.49 \| 12.86±0.50 \| 12.78±0.54 \| 12.59±0.06 B \| 13.63±0.47 A \| 12.66±0.60 B \| 12.79±0.43 B \| \| I3 \| 13.58±1.95 \| 12.83±1.60 \| 15.18±0.92 A \| 14.08±0.06 B \| 11.53±0.47 C \| 11.06±0.04 b \| 13.55±1.26 a \| 11.50±0.67 ab \| 13.71±1.77 a \| \| **Wet gluten content (%)** \| I1 \| 39.18±3.01 \| 41.29±2.18 \| 39.87±2.80 A \| 41.31±2.32 A \| 36.69±1.88 B \| 34.60±0.92 C \| 40.79±1.78 A \| 36.35±1.18 BC \| 39.00±3.01 AB \| \| I3 \| 41.05±3.52 \| 40.59±2.78 \| 41.16±3.80 A \| 40.33±1.91 A \| 35.06±3.81 B \| 34.37±2.48 \| 38.92±4.44 \| 36.03±6.14 \| 38.60±3.84 \| \| **Dough development time (min)** \| I1 \| 2.85±0.24 \| 2.63±0.23 \| 2.58±0.22 b \| 2.83±0.23 ab \| 2.96±0.36 a \| 3.20±0.36 a \| 2.77±0.35 b \| 3.03±0.27 ab \| 2.73±0.28 b \| \| I3 \| 3.00±0.24 \| 2.78±0.21 \| 2.73±0.24 \| 2.97±0.18 \| 2.83±0.36 \| 3.13±0.32 a \| 2.74±0.24 b \| 3.10±0.29 a \| 2.76±0.27 b \| \| **Dough stability time (min)** \| I1 \| 3.37±0.71 \| 3.02±0.60 \| 2.59±0.20 B \| 3.68±0.39 A \| 3.62±0.63 A \| 4.37±0.49 A \| 3.02±0.64 C \| 3.72±0.54 AB \| 3.27±0.57 BC \| \| I3 \| 3.65±0.52 a \| 2.92±0.54 b \| 2.76±0.41 B \| 3.57±0.55 A \| 3.61±0.78 A \| 4.63±0.49 A \| 2.97±0.56 C \| 4.00±0.60 B \| 3.18±0.53 C \| \| **Stretch area (cm^2^)** \| I1 \| 50.13±12.57 \| 43.13±9.89 \| 37.42±7.96 C \| 53.50±7.31 B \| 59.25±8.03 A \| 71.50±2.89 A \| 42.33±11.15 C \| 58.50±3.63 B \| 52.13±9.65 B \| \| I3 \| 54.25±11.95 a \| 44.06±10.43 b \| 38.33±6.12 C \| 56.58±8.38 A \| 49.75±8.34 B \| 63.50±5.74 A \| 43.58±10.52 B \| 48.75±4.89 B \| 48.58±9.72 B \| \| **Maximum resistance (EU)** \| I1 \| 244.13±19.01 \| 224.94±27.10 \| 226.75±27.86 B \| 235.92±24.26 B \| 284.96±38.15 A \| 336.25±12.01 A \| 215.92±19.56 D \| 295.13±25.83 B \| 253.92±27.19 C \| \| I3 \| 240.88±21.18 A \| 215.56±19.63 B \| 209.50±15.05 b \| 238.50±20.89 a \| 246.13±44.87 a \| 328.75±16.96 A \| 214.58±24.91 C \| 251.63±19.45 B \| 224.17±19.77 C \| \| **Tractility (mm)** \| I1 \| 140.25±22.44 \| 136.94±21.82 \| 120.42±8.96 C \| 155.67±14.72 A \| 138.75±9.10 B \| 143.50±11.82 \| 139.67±22.16 \| 132.15±6.20 \| 139.00±16.28 \| \| I3 \| 156.75±16.93 a \| 139.31±16.02 b \| 131.00±11.90 B \| 159.25±10.14 A \| 136.04±14.44 B \| 126.00±6.68 B \| 139.08±13.16 AB \| 128.50±9.34 B \| 147.79±17.84 A \| \| **Zeleny sedimentation value /mL** \| I1 \| 24.21±1.38 \| 24.94±1.73 \| 23.54±0.62 C \| 25.84±1.48 A \| 24.37±0.88 B \| 23.40±1.15 \| 25.36±1.95 \| 24.78±0.61 \| 24.22±0.83 \| \| I3 \| 25.82±1.48 \| 24.10±2.07 \| 22.96±1.31 B \| 26.39±0.63 A \| 21.13±0.88 C \| 22.43±0.25 \| 22.70±2.62 \| 21.02±0.15 \| 23.71±2.51 \| |
| The lowercase letter after the peer average indicates P<0.05, and the uppercase letter indicates P<0.01. I1: regime once at overwintering stage, I3: irrigation three times at overwintering, jointing, and filling stages. |
