# Supplementary material for: Effects of high-molecular-weight glutenin subunit on hard-steamed bread quality
Source: Front Genet. 2024 Oct 3;15:1473518. doi: 10.3389/fgene.2024.1473518 (PMC11484002; doi:10.3389/fgene.2024.1473518)
Supplement: Supplementary file 2 [file DataSheet1.docx]

Effects of high-molecular-weight glutenin subunit on hard-steamed bread quality

**Jing Zhao^1^**^†^**^,2^**, **Tianyi Wang^1^**^†^**^,2^**, **Hui Chen^1,2^**, **Jiajia Zhao^1^**, **Ling Qiao ^1^**, **Bangbang Wu^1^**, **Yuqiong Hao^1^**, **Chuan Ge^1^**, **Juanling Wang^2^**, **Zhiwei Feng^2*^**, **Xianghai Meng^3*^**

^1^ Institute of Wheat Research, Key Laboratory of Sustainable Dryland Agriculture (Co-construction by Ministry and Province) Ministry of Agriculture and Rural Affairs Shanxi Agricultural University, Linfen, China

^2^ College of Agriculture, Shanxi Agricultural University, Taigu, China

^3^ Dryland Farming Institute, Hebei Academy of Agriculture and Forestry Sciences

*** Correspondence:**

Zhiwei Feng and Xianghai Meng

E-mail address: zhiweifeng@126.com; mengxianghai5229@163.com

| 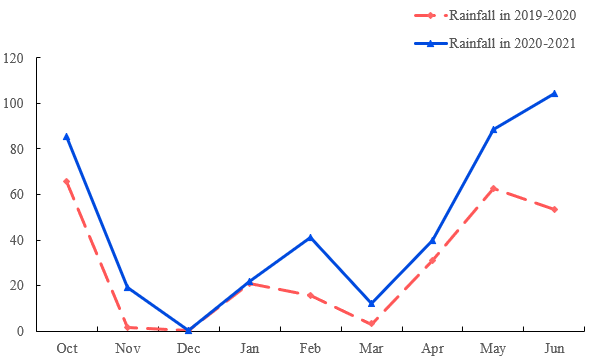 |
| --- |
| **Supplemental Figure 1** Precipitation during the growth period in 2019-2020 and 2020-2021 |

|  |
| --- |
| **Supplemental Figure 2** SDS-PAGE of HMW-GS in DHs |
| M1: Chinese Spring (N, 7+8, 2+12); M2: Shiluan02-1 (1,7+9,5+10); 1: Linfen5064 (1, 7+8, 2+12); 2: Nongda3338 (N, 6+8 5+12); 3: Jinmai47 (N, 7+9, 2+12); 4: Jinmai84 (1, 7+9, 5+10); 5~11: partial lines of DHs |

|  |
| --- |
| **Supplemental Figure 3** Correlation between steamed bread quality traits and rheological properties of dough |
| X1-X16 represents protein content, wet gluten content, dough development time, stability time, Zeleny sedimentation value, stretch area, tractility, maximum resistance, volume, specific volume, adhesion, cohesiveness, elasticity, glueyness, chewiness and hardness, respectively. |
